# Over 100-THz bandwidth selective difference frequency generation at LaAlO_3_/SrTiO_3_ nanojunctions

**DOI:** 10.1038/s41377-019-0135-0

**Published:** 2019-02-27

**Authors:** Lu Chen, Erin Sutton, Hyungwoo Lee, Jung-Woo Lee, Jianan Li, Chang-Beom Eom, Patrick Irvin, Jeremy Levy

**Affiliations:** 1 Department of Physics and Astronomy, University of Pittsburgh, Pittsburgh, PA 15260 USA; 2 Pittsburgh Quantum Institute, Pittsburgh, PA 15260 USA; 3 Department of Materials Science and Engineering, University of Wisconsin-Madison, Madison, WA 53706 USA

**Keywords:** Optoelectronic devices and components, Nonlinear optics, Sub-wavelength optics, Terahertz optics

## Abstract

The ability to combine continuously tunable narrow-band terahertz (THz) generation that can access both the far-infrared and mid-infrared regimes with nanometer-scale spatial resolution is highly promising for identifying underlying light-matter interactions and realizing selective control of rotational or vibrational resonances in nanoparticles or molecules. Here, we report selective difference frequency generation with over 100 THz bandwidth via femtosecond optical pulse shaping. The THz emission is generated at nanoscale junctions at the interface of LaAlO_3_/SrTiO_3_ (LAO/STO) that is defined by conductive atomic force microscope lithography, with the potential to perform THz spectroscopy on individual nanoparticles or molecules. Numerical simulation of the time-domain signal facilitates the identification of components that contribute to the THz generation. This ultra-wide-bandwidth tunable nanoscale coherent THz source transforms the LAO/STO interface into a promising platform for integrated lab-on-chip optoelectronic devices with various functionalities.

## Tunable terahertz on a chip scale

A nanojunction structure developed by researchers in the US can generate tunable terahertz radiation over a wide bandwidth. Terahertz radiation, occupying the upper end of microwave and lower end of infrared, causes atoms and molecules to resonate, revealing useful information about the physical structure of matter. However, terahertz radiation is difficult to generate. Lu Chen and co-workers in Jeremy Levy’s group at the University of Pittsburgh used an atomic force microscope to create nano-sized junctions between lanthanum aluminate and strontium titanate. By shining two frequencies of laser light onto a voltage-biased nanojunction, they could produce radiation at the difference of the two frequencies as high as 100 terahertz. Because the nanojunction can both generate and detect terahertz radiation, it could be used to manipulate and observe nanoscale objects such as quantum dots or individual molecules.

## Introduction

Electromagnetic waves at terahertz (THz) frequencies enable resonant interactions with matter through various intrinsic low-energy excitations, thereby revealing information that is related to the lattice, charge, and spin degrees of freedom. In the past few decades, extensive research efforts have focused on developing narrow-band THz sources in both far-infrared (<10 THz)^1^ and mid-infrared (10–100 THz)^2^ regimes, due to their potential to provide insight into the fundamental physics of matter via selective excitations of various resonances. For many applications, including spectroscopy^3^, inspection^4^, communication^5^, and coherent control^6^, tunability of the narrow-band THz emission is required. In addition, THz techniques are often limited in terms of spatial resolution^7,8^. Owing to the relatively long wavelength of the THz field, diffraction usually limits the spatial resolution to the order of 10–100 µm, thereby making it difficult to resolve features that are substantially below this scale. Several techniques have been proposed for realizing nanometer-scale spatial resolution, such as combining THz emission with scattering-type near-field scanning optical microscopy^9^ or scanning tunneling microscopy^10^. However, a continuously tunable, quasi-monochromatic THz source that can cover both the far- and mid-infrared regimes with sub-10-nm spatial resolution is not currently available.

Here, we report >100 THz bandwidth selective difference frequency generation at LaAlO_3_/SrTiO_3_ (LAO/STO) nanojunctions via femtosecond optical pulse shaping. Selected frequency components of a sub-7 fs ultrafast pulse are mixed at the nanojunction through the third-order nonlinear effect in STO^11^ and their frequency differences result in narrow-band THz emission. By controlling the selected frequency components, the frequency of the narrow-band THz emission can be tuned from the far-infrared to the mid-infrared regime. The spatial resolution of this THz source is determined by the nanojunction size and is typically approximately 10 nm but can be as small as 2 nm^11,12^, thereby realizing an ultra-broad-bandwidth, continuously tunable, quasi-monochromatic THz source with a spatial resolution that is comparable to a single nanoparticle or even a single molecule^13^.

## Results

**Fig. 1:**
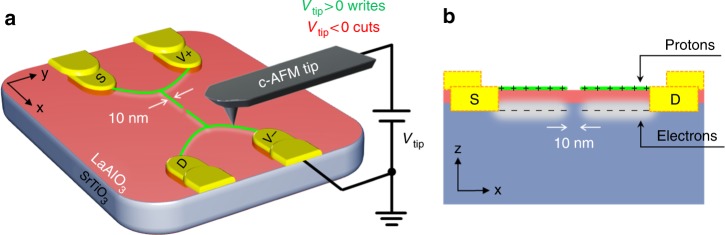
Schematic diagram of the four-terminal nanojunction device at the interface of LaAlO_3_/SrTiO_3_ (LAO/STO) for selective difference frequency generation. **a** Conductive atomic force microscope (c-AFM) lithography. Gold electrodes are patterned via conventional photolithography to form direct contacts with the LAO/STO interface. The green wires represent the designed device geometry. A positively biased AFM tip writes the conducting nanowires in contact mode, while a negatively biased AFM tip creates a nanojunction by cutting across the nanowire. **b** A side view of the sample shows that the c-AFM-lithography-defined device is located at the interface of the LAO/STO heterostructure. Both the nanowires and the nanojunction have a spatial confinement of approximately 10 nm. The dimensions are not to scale

The LAO/STO samples have an LAO layer thickness of 3.4 unit cells, which is just below the critical thickness^14^ of the metal-insulator transition, thereby resulting in an insulating interface. The LAO/STO nanojunctions are created via conductive atomic force microscope (c-AFM) lithography^12^, as illustrated in Fig. 1. A positively biased AFM tip is scanned along a line in contact over the LAO surface to locally charge the LAO surface with protons^15,16^, which attract electrons to the buried interface to form a conducting nanowire that has a typical width of 10 nm. A negatively biased AFM tip is scanned over the conducting regions to remove the adsorbed protons, thereby restoring the interface to an insulating state. Of particular relevance to this work is the “nanojunction” pattern, in which a nanowire is created with a nanoscale (~10 nm) insulating gap (Fig. 1b).

A four-terminal structure with a nanojunction in the middle (Fig. 1a) is designed and created at the LAO/STO interface for the selective difference frequency THz generation, where electrodes, which are labeled *S* and *D* in the figure, are used to apply a DC bias voltage, which is denoted as *V*_*dc*_, across the nanojunction and two voltage-sensing electrodes (*V*^+^ and *V*^*−*^) are used to measure the photovoltage change that is induced by ultrafast laser pulses, which will be described in detail below. The four-terminal geometry provides an accurate measurement of the photoinduced voltage change across the nanojunction since any voltage drops in the leads, external wires or imperfect contacts are eliminated in this geometry.

**Fig. 2:**
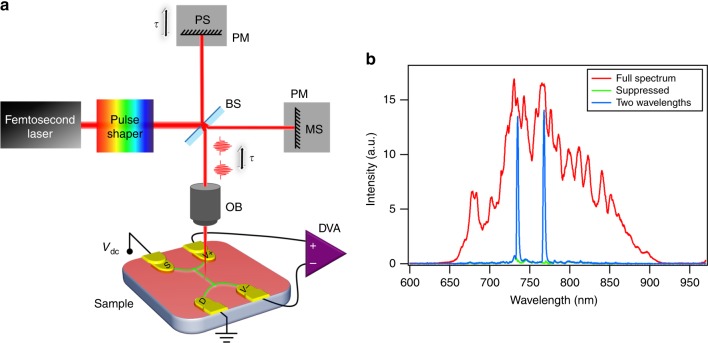
Optical setup and spectral amplitude control. **a** Schematic diagram of the optical setup. BS: beam splitter, PM: plane mirror, MS: mechanical stage, PS: piezoelectric stage, OB: objective, and DVA: differential voltage amplifier. The dimensions are not to scale. **b** Spectral amplitude control of the femtosecond optical pulse by the pulse shaper

Figure 2a shows the schematic drawing of the experimental setup. The ultrafast pulses from a sub-7-fs Ti: Sapphire oscillator (Spectra-Physics Rainbow 2 UHP) is directed into an optical pulse shaper that is based on a dual-mask spatial light modulator (SLM, Jenoptik SLM-S640d), where wavelengths are spatially separated by a grating and focused onto various pixels of the SLM. Both the amplitude and the phase of the ultrafast pulse can be controlled independently. Here, we focus on spectral amplitude control. After exiting the pulse shaper, the manipulated pulses are redirected to a compact Michelson interferometer that has two arms of approximately equal length. A p-polarized 50/50 ultrafast beam splitter (BS) splits the input pulses into two beams. The reflected beam is normally incident to a plane mirror (PM) that is mounted on a piezoelectric stage (PS), which serves as an optical delay line. The transmitted beam reflects off a plane mirror that is mounted on a mechanical stage, which enables coarse adjustment of the time delay. Both beams are recombined by the same beam splitter after normal reflection and subsequently focused onto the nanojunction by an objective (OB). During the measurement, the delay line is scanned continuously from negative to positive time delay values. A DC bias voltage (*V*_*dc*_ = −550 mV) is applied to electrode *S* through a 50-Ω-impedance analog output port, while electrode *D* is grounded. The photovoltage, which is the voltage difference, namely, Δ*V* = *V*^+^−*V*^−^, between the two voltage sensing electrodes, is measured and amplified by a differential voltage amplifier (DVA) with 1-MΩ input impedance and recorded as a function of the time delay, which is denoted as *τ*.

Figure 2b shows an example of pulse spectral amplitude control by the pulse shaper. The red curve represents the full spectrum from the Ti:Sapphire oscillator without any spectral amplitude manipulation. A broad spectrum that ranges from 650 nm to 920 nm is measured by a spectrometer. By applying an appropriate voltage to each pixel of the SLM, the output at all wavelengths can be efficiently suppressed (green curve). Then, we can select one, two or a few wavelengths to pass through the SLM, while keeping all the other wavelengths suppressed. The blue curve corresponds to a configuration in which light at 735 nm and 768 nm is allowed to pass through the SLM, while other wavelengths are suppressed.

**Fig. 3:**
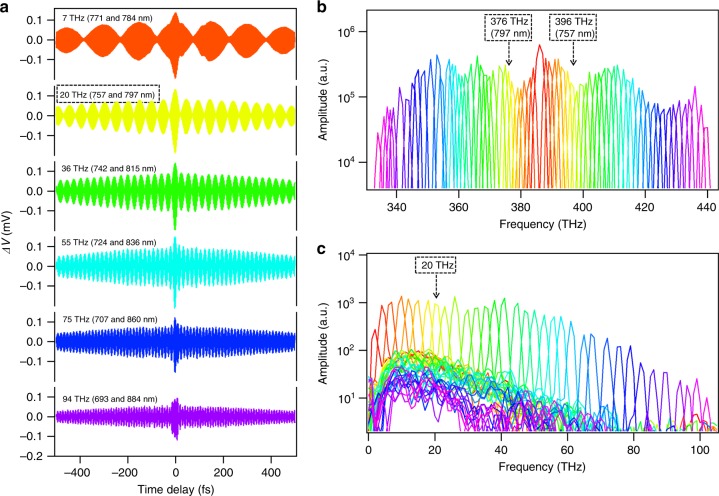
Over 100 THz ultra-broad-bandwidth selective difference frequency generation at the LAO/STO nanojunction. **a** Time-domain signals of six representative difference frequency generations. **b** Power spectra of time-domain signals that show 35 pairs of selected fundamental frequencies. **c** The 35 difference frequencies that are generated through the third-order nonlinear effect at the nanojunction that spans the entire far- to mid-infrared regime. All plots are color-coded

To demonstrate the ultra-broad-bandwidth selective difference frequency generation capability at the LAO/STO nanojunction, we perform nonlinear wavelength mixing experiments. We select 35 fundamental wavelength pairs with the frequency difference within each pair ranging from 2 THz to 106 THz. The total input excitation power is on the order of 100 µW. For each fundamental wavelength pair, the amplified photovoltage, namely, Δ*V*, is recorded as the optical time delay line varies from *τ* = −500 fs to+500 fs and the same measurement is repeated 40 times for averaging. Fig. 3a shows six representative averaged time-domain signals, with their difference frequencies and fundamental wavelength pairs labeled accordingly. The curves are distinguished by color and all plots in Fig. 3 share the same color correspondences. A constant background has been subtracted for each curve, which originates from the DC bias voltage and the persistent photoconductance by mid-gap states in STO^17^. A beating envelope is observed in each signal and the lower half of the envelope has a larger amplitude than the upper half. Power spectra (Fig. 3b and c) are calculated from the time-domain signals to identify the frequency components. Fig. 3b shows the frequencies of all 35 selected fundamental wavelength pairs, while Fig. 3c displays the 35 resulting selective difference frequencies that are generated at the LAO/STO nanojunction. For example, a fundamental wavelength pair of 757 nm (396 THz) and 797 nm (376 THz) is selected from the ultrafast pulse by the pulse shaper to generate a narrow-band emission at 20 THz. The corresponding time-domain signal is measured and plotted in Fig. 3a (yellow curve) and the fundamental wavelength pair is clearly observed in its power spectrum (at the arrowheads in Fig. 3b), which demonstrates that a 20 THz difference frequency is generated (as indicated in Fig. 3c). The measured linewidth of the narrow-band THz generation (~2 THz on average) in this configuration is limited by the spectral resolution of the SLM and the total travel range of the optical delay line (1 ps, which corresponds to 1 THz resolution).

## Discussion

Due to the large bandgaps for both LAO and STO, the input photon energies in the experiments are not sufficient for exciting valence electrons to the conduction band. Although there are mid-gap states in STO^18^, the reported lifetime for STO photoexcited carriers exceeds tens of nanoseconds under the current experimental conditions^19,20^, which is not sufficiently fast for generating ultra-broad-bandwidth THz fields or for detecting the generated THz field via photoconductive sampling. However, the corresponding nonlinear optical process (optical rectification) is able to generate a broadband THz field. The experiments are conducted at temperature *T* = 80 K. Below *T* = 105 K, bulk STO undergoes a cubic-to-tetragonal transition; however, the STO remains centrosymmetric, with a vanishing second-order susceptibility, which is denoted as $\chi ^{\left( 2 \right)}$. Even though the breaking of inversion symmetry at the interface of LAO/STO can produce a $\chi ^{\left( 2 \right)}$ response^21,22^, the 2D nature of the interface makes it unlikely for the second-order nonlinear effect to play a dominant role. In contrast, the third-order susceptibility, which is denoted as $\chi ^{\left( 3 \right)}$, is exceedingly large for bulk STO^23^. It has been experimentally demonstrated that the ultrafast photoconductive response at the LAO/STO nanojunction is DC electric field tunable and spatially confined to the region of the nanojunction^11^. These prior results suggest that the third-order nonlinear effect is the leading mechanism for wave mixing. The nanometer-scale dimension (~10 nm) of the nanojunction provides strong confinement of the DC bias field, thereby resulting in an intensity of 5.5 × 10^5^ V/cm for *V*_*dc*_ = −550 mV. In this sense, the third-order nonlinear process can also be viewed as a DC bias field-mediated second-order nonlinear process.

The time-varying optical field, namely, *E*_*opt*_, from the ultrafast pulses and the quasi-static bias field, namely, *E*_*bias*_, from the DC bias voltage interact at the LAO/STO nanojunction, thereby resulting in a change in the polarization, which is denoted as *P*, in STO:

1 $$P = \varepsilon _0\left( {\chi ^{(1)}E_{opt} + \chi ^{(3)}E_{bias}^2E_{opt} + \chi ^{(3)}E_{bias}E_{opt}^2 + \chi ^{(3)}E_{opt}^3} \right)$$

where $\varepsilon _0$ is the vacuum permittivity and $\chi ^{(1)}$ is the linear susceptibility of STO. Frequency components $\omega _1$ and $\omega _2$ in the optical fields mix and the resulting time-varying polarization induces a field, which offsets the applied DC electric field and further mixes with the bias field, the optical field and even with itself to produce a photoinduced voltage change at the difference frequency of $\omega _1 - \omega _2$ at the LAO/STO nanojunction.

**Fig. 4:**
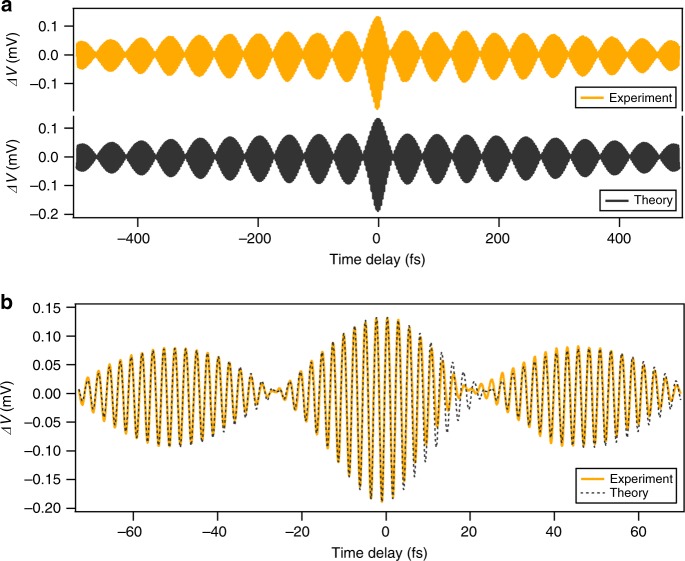
Comparison between the numerical simulation results and the measured time-domain signal. **a** Both the beating envelope and the asymmetry in the upper and lower amplitudes of the envelope are reproduced in the simulation plot. **b** A close-up of the measured and simulated time-domain signals near time delay τ = 0 shows good agreement between the two

To investigate the underlying physical mechanism, a numerical simulation of the measured time-domain signal has been performed (Fig. 4). The shape of the input pulse is approximated by a Gaussian:

2 $$e^{ - \left( {t/t_p} \right)^2}\cos \left( {\omega _ct} \right)$$

where *t*_p_ is the pulse width and $\omega _c$ is the central angular frequency of the pulse. Then, the simulated time-domain signal takes the form:

3 $$	\Delta V\left( \tau \right)\sim a\left(\vphantom{de^{ - \left( {\frac{\tau }{{t_p}}} \right)^2}} \left[ \cos \left( {\omega _1\tau } \right) + \cos \left( {\omega _2\tau } \right) \right] + b\left[ \cos \left( {2\omega _1\tau } \right)\right.\right. \\ 	\hskip 6pt \left.+ \cos \left( {2\omega _2\tau } \right) + 4\left( \cos \left[ {\left( {\omega _1 - \omega _2} \right)\tau } \right] + \cos \left[ {\left( {\omega _1 + \omega _2} \right)\tau } \right] \right) \right] \\ 	\hskip 6pt + \left[ce^{ - \frac{1}{2}\left( {\frac{\tau }{{t_p}}} \right)^2} + 4de^{ - \frac{3}{4}\left( {\frac{\tau }{{t_p}}} \right)^2}\right]\cos \left( {\omega _c\tau } \right) \\ 	\hskip 6pt \left.+ \, de^{ - \left( {\frac{\tau }{{t_p}}} \right)^2}\left[ 2 + \cos \left( {2\omega _c\tau } \right) \right] \right)$$

Here, *a,b,c* and *d* are fit coefficients (see Supplementary Information for a detailed derivation). In this simulation, $a \cong 4.33 \times 10^{ - 5}$ (V), $b = 0.018$, $c = 1.10$, and *d* = 0.154. The first term represents the linear response of the two selected frequencies at the LAO/STO nanojunction. The second term corresponds to the frequency mixing through the third-order nonlinear effect. The third and fourth terms are the induced linear and third-order nonlinear photoconductive response by the pulse at the nanojunction, respectively. These two terms exist because of the small nonvanishing fundamental pulse background (the baseline of the blue curve in Fig. 2b). In Fig. 4a, we compare the measured time-domain signal at a difference frequency of 20 THz (the yellow curve in Fig. 3a) with the results of the numerical simulation. Both the beating envelope and the asymmetry in the upper and lower amplitude of the envelope are reproduced. The overall decay of the signal amplitude is due to the finite width of the two selected fundamental wavelengths. Fig. 4b shows a magnified view of the measured signal and the simulated response near *τ* = 0; satisfactory agreement is observed between the two. The unequal amplitudes of the lower and upper envelopes are a result of the nonlinear process that produces the THz response. The fast oscillation with a beating envelope mostly originates from the superposition of the two fundamental frequencies. Discrepancies between the measured signal and the simulated signal are most visible at the node (near *τ* = 20fs) and are attributed to imperfect alignment of the two beams during the movement of the optical time delay line. This theoretical model also predicts that the fundamental signal amplitude depends linearly on the laser excitation power, while the THz amplitude depends on the square of the laser power (see Supplementary Information for more details); these predictions are also supported by experimental results (Figure S1). In addition to the main nonresonant DC bias field-mediated nonlinear wave mixing process, other responses could contribute to the measured signal. For example, the photoexcited free carriers from the mid-gap states could introduce near-resonant structure into the response. Nonetheless, the good agreement between the simulation results (fit to Eq. (3)) and the experimental data demonstrates that these responses are unlikely to play a dominant role.

Compared to other ultra-broad-bandwidth THz sources, such as free-electron lasers^24^ and nonlinear crystals, such as GaSe crystals^25^, the LAO/STO nanojunctions are easy to fabricate and reconfigurable and do not rely on phase matching due to the extremely small dimensions of the device. Here, the bandwidth of the THz emission is not restricted by the material; it is limited only by the spectral bandwidth of the ultrafast pulses. Moreover, the LAO/STO nanojunctions naturally yield a high spatial resolution. By simply drop-casting the target nanoscale objects onto the LAO/STO surface and creating a nanojunction in the vicinity of a single particle or molecule, individual nanoscale objects can be addressed independently, thereby offering insights that would otherwise be inaccessible via averaging over the ensemble. Spatial mapping of arbitrary substrates is also possible by scanning an LAO/STO nanojunction device in close proximity to the sample (or the other way around). Variations on the sample surface lead to modifications in the interaction among various fields at the nanojunction, which can be reflected by the measured photoinduced voltage change, with a spatial resolution that is determined by the nanojunction size. However, the extremely small size of the nanojunction results in a small amplitude of the THz emission. THz fields that are generated at LAO/STO nanojunctions are mostly in the near-field regime, which is a regime that is home to many interesting short-range interactions. In this work, we only control the amplitude of the input ultrafast pulse. Full use of the dual-mask SLM, via which both amplitude and phase modulation can be realized, can enable the realization of THz waveforms of arbitrary shape for future applications.

In conclusion, we have demonstrated over-100-THz-bandwidth selective difference frequency generation at LAO/STO nanojunctions that spans the entire far-infrared to mid-infrared regime via femtosecond optical pulse shaping. The ultrabroad tunability, combined with an exceptional spatial precision of 10 nm, is highly promising for exploring the fundamental physics of single nanoscale objects such as quantum dots, nanoparticles or individual molecules. The low optical excitation power imposes minimal heating or other adverse effects on the analyte. The LAO/STO nanojunction serves as both a generator and a detector of THz emission^11^. By writing two similar nanojunctions adjacent to each other, the second nanojunction can be used to detect the THz field that is generated from the first nanojunction, without the implication of beating signals. In this way, it is possible to realize both generation and detection of tunable ultra-broad-bandwidth THz fields in a micrometer-scale area. Numerous nanoelectronic devices have already been realized at the LAO/STO interface, such as photodetectors^17^ and field-effect transistors^26^. Combining the versatility of the LAO/STO nanodevices with tunable THz functionality opens a new pathway toward the realization of integrated lab-on-chip optoelectronic devices.

## Materials and methods

### Sample growth and patterning

The LAO/STO samples are grown via pulsed laser deposition. A thin film (3.4 unit cells) of LAO is deposited epitaxially on the (001) TiO_2_-terminated STO substrate at 550 °C and an oxygen pressure of 10^–3^ mbar, with its thickness monitored in situ via high-pressure reflection high-energy electron diffraction (RHEED). Additional details on the growth method are provided elsewhere^27^. Electrical contacts to the interface are fabricated via conventional photolithography, where predefined regions are etched via Ar^+^ ion milling (25 nm) and filled with Ti/Au (4 nm/25 nm). A second layer of Ti/Au is added on top of the LAO surface for wire bonding.

### c-AFM lithography

Conductive atomic force microscope lithography is performed using an Asylum Research Cypher AFM. The DC writing voltage is amplified by a high-voltage amplifier (Falco Systems WMA-02) and applied to the AFM tip (highly doped silicon) through a 1 GΩ series resistor. The interface of LAO/STO is kept grounded during c-AFM lithography. The devices are written under ambient conditions with a relative humidity of approximately 30–40%.

## Supplementary information

Supplementary Information
