# Supplementary material for: Over 100-THz bandwidth selective difference frequency generation at LaAlO3/SrTiO3 nanojunctions
Source: Light Sci Appl. 2019 Feb 27;8:24. doi: 10.1038/s41377-019-0135-0 (PMC6393436; doi:10.1038/s41377-019-0135-0)
Supplement: Supplementary file 1 — Supplementary Information [file 41377_2019_135_MOESM1_ESM.docx]

Supplementary Information

Over 100-THz Bandwidth Selective Difference Frequency Generation at LaAlO_3_/SrTiO_3_ Nanojunctions

*Lu Chen,^1,2^ Erin Sutton,^1,2^ Hyungwoo Lee,^3^ Jung-Woo Lee,^3^ Jianan Li,^1,2^ Chang-Beom Eom,^3^ Patrick Irvin,^1,2^ and Jeremy Levy^1,2*^*

^1^Department of Physics and Astronomy, University of Pittsburgh, Pittsburgh, Pennsylvania 15260, USA

^2^Pittsburgh Quantum Institute, Pittsburgh, Pennsylvania 15260, USA

^3^Department of Materials Science and Engineering, University of Wisconsin-Madison, Madison, Wisconsin 53706, USA

*^*^*Correspondence: jlevy@pitt.edu

**Theoretical model for the time domain signal**

The electric field of the fundamental wavelength pair selected by the pulse shaper can be expressed as

|  | $E_{input}=E_{1}\cos\left( \omega_{1}t \right)+E_{2}\cos\left( \omega_{2}t \right)$ | (S1) |
| --- | --- | --- |

where $E_{1}$ and $E_{2}$ are the amplitude, and $\omega_{1}$ and $\omega_{2}$ are the angular frequency of the plane wave for the two fundamental wavelengths, respectively. The intensity of this input field $E_{input}$ is then divided equally by a 50/50 ultrafast beam splitter. An additional time delay 𝜏 is added to the reflected beam by scanning the piezoelectric stage, compared to the transmitted beam. The electric field for the transmitted beam $E_{tran}$ and the reflected beam $E_{refl}$ are thus given by

|  | $E_{tran}=\frac{1}{\sqrt{2}}\left[ E_{1}\cos\left( \omega_{1}t \right)+E_{2}\cos\left( \omega_{2}t \right) \right]$ | (S2) |
| --- | --- | --- |
|  | $E_{refl}=\frac{1}{\sqrt{2}}\left( E_{1}\cos{[\omega}_{1}\left( t-\tau\right)]+E_{2}\cos{[\omega}_{2}\left( t-\tau\right)] \right)$ | (S3) |

The two beams are re-combined by the same beam splitter, and focused onto the LaAlO_3_/SrTiO_3_ (LAO/STO) nanojunction, yielding an optical electric field $E_{opt}$ in the following form

|  | $E_{opt}=\frac{1}{2}\left( E_{1}\cos\left( \omega_{1}t \right)+E_{2}\cos\left( \omega_{2}t \right)+E_{1}\cos{[\omega}_{1}\left( t-\tau\right)]+E_{2}\cos{[\omega}_{2}\left( t-\tau\right)] \right)$ | (S4) |
| --- | --- | --- |

A DC bias voltage is applied across the nanojunction, which can be described as a quasi-static local field $E_{bias}$. The optical field and the bias field interact at the nanojunction, resulting in a change in the polarization *P* in STO:

|  | $P=\varepsilon_{0}\left( \chi^{(1)}E_{opt}+\chi^{(3)}E_{bias}^{2}E_{opt}+\chi^{(3)}E_{bias}E_{opt}^{2}+\chi^{(3)}E_{opt}^{3} \right)$ | (S5) |
| --- | --- | --- |

where $\varepsilon_{0}$ is vacuum permittivity, $\chi^{(1)}$ and $\chi^{(3)}$ are the linear and third-order nonlinear susceptibility of STO, respectively. The second-order nonlinear response is neglected, for reasons that are described in the main text. This time-varying polarization generates an induced electric field $E_{induced}$, which offsets the photovoltage across the nanojunction. In addition, the induced field can also mix with the bias field, optical field and itself, further modulating the polarization in STO. The resulting photo-induced voltage change $\Delta V_{selected}\left( \tau\right)$ at the nanojunction for the selected wavelength pair thus takes the form

|  | $\Delta V_{selected}\left( \tau\right) \sim E_{induced}+\chi^{(1)}E_{induced}+\chi^{(3)}E_{bias}^{2}E_{induced}+\chi^{(3)}E_{bias}E_{opt}E_{induced}+\chi^{(3)}E_{bias}E_{induced}^{2}+\chi^{(3)}E_{opt}^{2}E_{induced}+\chi^{(3)}E_{opt}E_{induced}^{2}+\chi^{(3)}E_{induced}^{3}$ | (S6) |
| --- | --- | --- |

We ignore $\left( \chi^{(3)} \right)^{3}$ terms. And due to the slow sample response time compared to the optical frequencies, terms containing $\omega_{1}t$ or $\omega_{2}t$ vanish, leading to

|  | $\Delta V_{selected}\left( \tau\right) \sim\frac{\chi^{\left( 3 \right)}E_{bias}}{64}\left( 4E_{1}^{2}\left[ 4+6\chi^{\left( 3 \right)}E_{1}^{2}+12\chi^{\left( 3 \right)}E_{2}^{2}+8\chi^{\left( 3 \right)}E_{bias}^{2}+\left( \chi^{\left( 1 \right)} \right)^{2}\left( 4+9\chi^{\left( 3 \right)}E_{1}^{2}+18\chi^{\left( 3 \right)}E_{2}^{2} \right)+4\chi^{\left( 1 \right)}\left( 2+3\chi^{\left( 3 \right)}E_{1}^{2}+6\chi^{\left( 3 \right)}E_{2}^{2}+2\chi^{\left( 3 \right)}E_{bias}^{2} \right) \right]\cos\left( \omega_{1}\tau\right)+4E_{2}^{2}\left[ 4+6\chi^{\left( 3 \right)}E_{2}^{2}+12\chi^{\left( 3 \right)}E_{1}^{2}+8\chi^{\left( 3 \right)}E_{bias}^{2}+\left( \chi^{\left( 1 \right)} \right)^{2}\left( 4+9\chi^{\left( 3 \right)}E_{2}^{2}+18\chi^{\left( 3 \right)}E_{1}^{2} \right)+4\chi^{\left( 1 \right)}\left( 2+3\chi^{\left( 3 \right)}E_{2}^{2}+6\chi^{\left( 3 \right)}E_{1}^{2}+2\chi^{\left( 3 \right)}E_{bias}^{2} \right) \right]\cos\left( \omega_{2}\tau\right)+3\left[ 2+4\chi^{\left( 1 \right)}+3\left( \chi^{\left( 1 \right)} \right)^{2} \right]\chi^{\left( 3 \right)}\left[ E_{1}^{4}\cos\left( 2\omega_{1}\tau\right)+E_{2}^{4}\cos\left( 2\omega_{2}\tau\right) \right]+12E_{1}^{2}E_{2}^{2}\left[ 2+4\chi^{\left( 1 \right)}+3\left( \chi^{\left( 1 \right)} \right)^{2} \right]\chi^{\left( 3 \right)}\left( \cos\left[ \left( \omega_{1}-\omega_{2} \right)\tau\right]+\cos\left[ \left( \omega_{1}+\omega_{2} \right)\tau\right] \right) \right)$ | (S7) |
| --- | --- | --- |

The small non-vanishing fundamental pulse background after the pulse shaper also contributes to the measured photo-induced voltage change. We approximate the input pulse shape as a Gaussian:

|  | $E_{input}^{Pulse}\left( t \right)=E_{0}e^{-\left( t/{t_{p}} \right)^{2}}\cos\left( \omega_{c}t \right)$ | (S8) |
| --- | --- | --- |

Where $t_{p}$ is the pulse width, $E_{0}$ is the amplitude and $\omega_{c}$ is the central angular frequency of the pulse wave. Similarly, the focused pulse optical field at the nanojunction is given by

|  | $E_{opt}^{pulse}=\frac{E_{0}}{2}\left( e^{-\left( \frac{t}{t_{p}} \right)^{2}}\cos\left( \omega_{c}t \right)+e^{-\left( \frac{t-\tau}{t_{p}} \right)^{2}}\cos{[\omega}_{c}\left( t-\tau\right)] \right)$ | (S9) |
| --- | --- | --- |

Since the material response time is much longer than the pulse duration, an integral of t from $-\infty$ to $\infty$ is needed to derive the pulse-induced photovoltage change $\Delta V_{pulse}\left( \tau\right)$ across the nanojunction:

|  | $\Delta V_{pulse}\left( \tau\right)\sim\int_{-\infty}^{\infty} \left( E_{induced}+\chi^{(1)}E_{induced}+\chi^{(3)}E_{bias}^{2}E_{induced}+\chi^{(3)}E_{bias}E_{opt}^{pulse}E_{induced}+\chi^{(3)}E_{bias}E_{induced}^{2}+\chi^{(3)}\left( E_{opt}^{pulse} \right)^{2}E_{induced}+\chi^{(3)}E_{opt}^{pulse}E_{induced}^{2}+\chi^{(3)}E_{induced}^{3} \right)dt$ | (S10) |
| --- | --- | --- |

Ignore $\left( \chi^{(3)} \right)^{3}$ and higher order terms. Terms containing $e^{-\left( \omega_{c}t_{p} \right)^{2}}$ can also be ignored owing to their extreme small values (${-\left( \omega_{c}t_{p} \right)}^{2}\cong-590)$. The computed $\Delta V_{pulse}\left( \tau\right)$ then reads

|  | $\Delta V_{pulse}\left( \tau\right)\sim\frac{\chi^{(3)}E_{0}^{2}E_{bias}}{128}t_{p}\sqrt{\pi}\left( \left( 12E_{0}^{2}\left[ 2+\chi^{\left( 1 \right)}\left( 4+3\chi^{\left( 1 \right)} \right) \right]\chi^{\left( 3 \right)}e^{-\frac{3}{4}\left( \frac{\tau}{t_{p}} \right)^{2}}+16\sqrt{2}\left( 1+\chi^{\left( 1 \right)} \right)\left( 1+\chi^{\left( 1 \right)}+2\chi^{\left( 3 \right)}E_{bias}^{2} \right)e^{-\frac{1}{2}\left( \frac{\tau}{t_{p}} \right)^{2}} \right)\cos\left( \omega_{c}\tau\right)+3E_{0}^{2}\left[ 2+\chi^{\left( 1 \right)}\left( 4+3\chi^{\left( 1 \right)} \right) \right]\chi^{\left( 3 \right)}e^{-\left( \frac{\tau}{t_{p}} \right)^{2}}[2+\cos\left( 2\omega_{c}\tau\right)] \right)$ | (S11) |
| --- | --- | --- |

The measured photo-induced voltage change $\Delta V\left( \tau\right)$ across the LAO/STO nanojunction is the sum of both $\Delta V_{selected}\left( \tau\right)$ and $\Delta V_{pulse}\left( \tau\right)$:

|  | $\Delta V\left( \tau\right)=\Delta V_{selected}\left( \tau\right)+\Delta V_{pulse}\left( \tau\right)$ | (S12) |
| --- | --- | --- |

For simplicity, we further assume $E_{2}\cong E_{1}$, and rewrite Eq. (S7) and Eq. (S11) into

|  | $\Delta V\left( \tau\right) \sim a\left( \left[ \cos\left( \omega_{1}\tau\right)+\cos\left( \omega_{2}\tau\right) \right]+b\left[ \cos\left( 2\omega_{1}\tau\right)+\cos\left( 2\omega_{2}\tau\right)+4\left( \cos\left[ \left( \omega_{1}-\omega_{2} \right)\tau\right]+\cos\left[ \left( \omega_{1}+\omega_{2} \right)\tau\right] \right) \right]+[ce^{-\frac{1}{2}\left( \frac{\tau}{t_{p}} \right)^{2}}+4 de^{-\frac{3}{4}\left( \frac{\tau}{t_{p}} \right)^{2}}]cos \left( \omega_{c}\tau\right)+de^{-\left( \frac{\tau}{t_{p}} \right)^{2}}\left[ 2+\cos\left( 2\omega_{c}\tau\right) \right] \right)$ | (S13) |
| --- | --- | --- |

using following abbreviations

|  | $a=\frac{1}{16}[4+18\chi^{\left( 3 \right)}E_{1}^{2}+8\chi^{\left( 3 \right)}E_{bias}^{2}+\left( \chi^{\left( 1 \right)} \right)^{2}\left( 4+27\chi^{\left( 3 \right)}E_{1}^{2} \right)+4\chi^{\left( 1 \right)}(2+9\chi^{\left( 3 \right)}E_{1}^{2}+2\chi^{\left( 3 \right)}E_{bias}^{2})]\chi^{(3)}E_{1}^{2}E_{bias}$ | (S14) |
| --- | --- | --- |
|  | $b=\frac{3}{64a}\left[ 2+\chi^{\left( 1 \right)}\left( 4+3\chi^{\left( 1 \right)} \right) \right]\left( \chi^{\left( 3 \right)} \right)^{2}E_{1}^{4}E_{bias}$ | (S15) |
|  | $c=\frac{1}{8a}t_{p}\sqrt{2\pi}\left( 1+\chi^{\left( 1 \right)} \right)\left( 1+\chi^{\left( 1 \right)}+2\chi^{\left( 3 \right)}E_{bias}^{2} \right)\chi^{(3)}E_{0}^{2}E_{bias}$ | (S16) |
|  | $d=\frac{3}{128a}t_{p}\sqrt{\pi}\left[ 2+\chi^{\left( 1 \right)}\left( 4+3\chi^{\left( 1 \right)} \right) \right]\left( \chi^{\left( 3 \right)} \right)^{2}E_{0}^{4}E_{bias}$ | (S17) |

A nonlinear least-squares fit is performed to obtain the initial fitting parameters. The initial values for *a-d* and their corresponding standard errors are listed below

|  | Estimate | Standard Error |
| --- | --- | --- |
| *a* | -0.0000425596 | 3.46618×10^-8^ |
| *b* | 0.0128076 | 0.000221672 |
| *c* | 0.887046 | 0.0123936 |
| *d* | 0.147767 | 0.00276426 |

A slight fine-tuning of *a-d* is then performed manually around the initial values to better reproduce the experimental data, and final values used for the simulation in the main text are

|  | *a* | *b* | *c* | *d* |
| --- | --- | --- | --- | --- |
| Coefficient | 4.33×10^-5^ | 0.018 | 1.1 | 0.154 |

**Dependence of the signal amplitude on the laser power**

Here, the full wavelength of the laser is used to excite the LAO/STO nanojunction. The laser power is measured with a power meter calibrated at 800 nm. Both the fundamental and the THz amplitude are plotted as a function of the laser power in Figure S1. The fundamental amplitude at each laser power is calculated by integrating the power spectrum amplitudes from 335 THz to 445 THz, and the THz amplitude is integrated from 0 THz to 100 THz. The fundamental amplitude mostly follows a linear dependence on the laser excitation power, while the THz amplitude depends on the square of the laser power. Both results agree very well with the theoretical model (second half of Eq. (S13), with coefficients written out by Eq. (S16) and (S17)). Deviations of the fundamental (THz) signal from the linear (square) dependence at low laser powers are attributed to systematic offsets in the power reading at the lowest intensities. Another quantity that can be analyzed is the ratio of the THz response to the fundamental. This ratio is defined as $Ratio={A_{THz}}/{A_{Fund}}$, where $A_{THz}$ and $A_{Fund}$ are the integrated THz and fundamental amplitude in arbitrary units, respectively. The $Ratio$ (inset of Figure S1) shows a linear dependence on the laser excitation power, as expected.


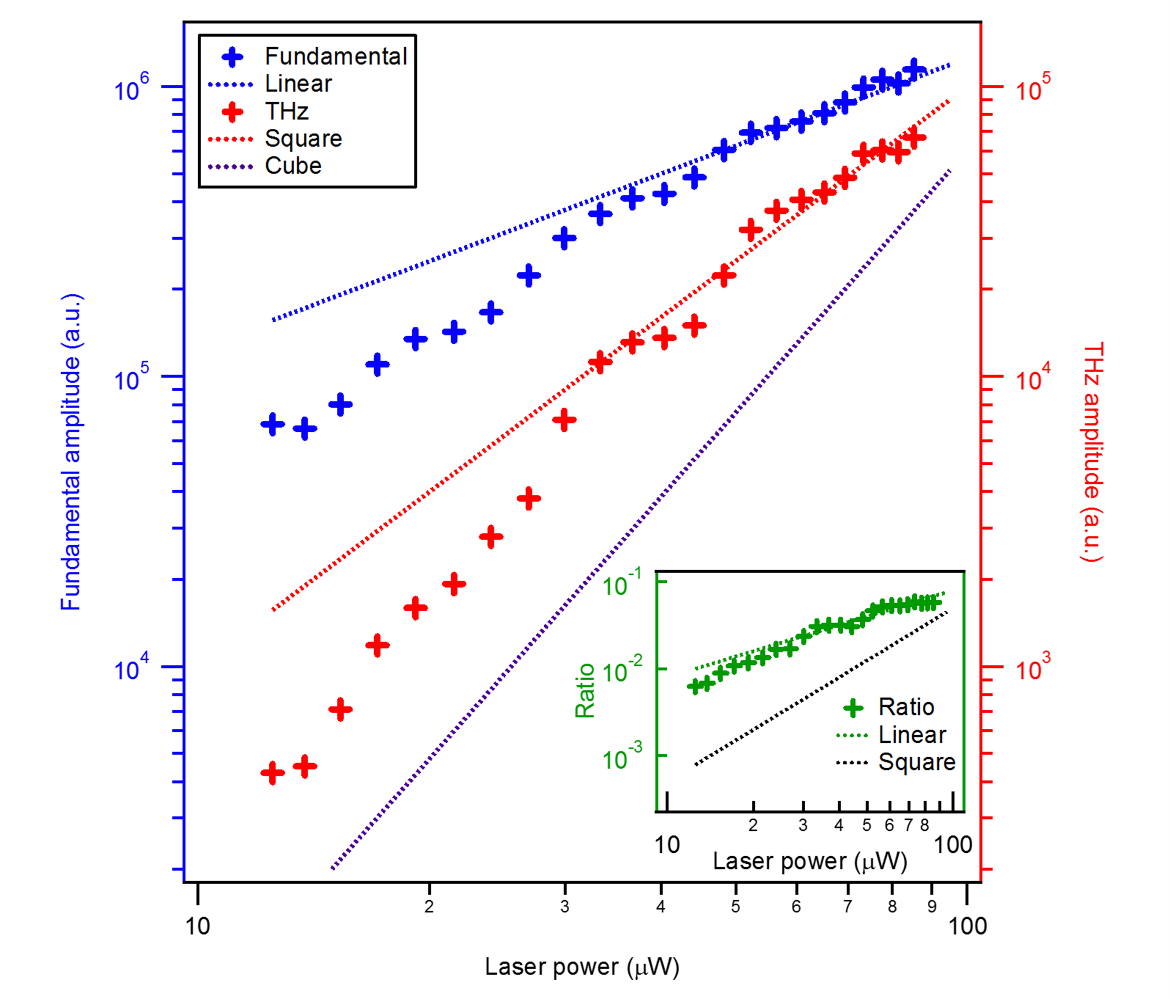


**Figure S1.** Dependence of the fundamental and the THz spectral amplitude on the laser excitation power. The graph is plotted on a log-log scale. Blue markers are the integrated fundamental power spectrum amplitudes (from 335 THz to 445 THz). The blue dotted line shows a linear dependence to guide the eye, with $A_{Fund}=12500P$, where $A_{Fund}$ is the integrated fundamental amplitude in arbitrary units and *P* is the laser power. Red markers are the integrated THz power spectrum amplitudes (from 0 THz to 100 THz). The red dotted line shows a square dependence to guide the eye, with $A_{THz}=10P^{2}$, where $A_{THz}$ is the integrated THz amplitude in arbitrary units. A cubic dependence (purple dotted line) is also shown for comparison, which takes the form of $0.06P^{3}$. Green markers in the inset are the ratios of the THz amplitude to the fundamental amplitude ($Ratio={A_{THz}}/{A_{Fund}}$) at different laser powers. The green dotted line shows a linear dependence to guide the eye, with $Ratio=0.0008P$. A square dependence (black dotted line) is also shown in the inset for comparison, which takes the form of $5\times{10}^{-6}P^{2}$.
